# Identification of Crucial Genes and Infiltrating Immune Cells Underlying Sepsis-Induced Cardiomyopathy via Weighted Gene Co-Expression Network Analysis

**DOI:** 10.3389/fgene.2021.812509

**Published:** 2021-12-24

**Authors:** Juexing Li, Lei Zhou, Zhenhua Li, Shangneng Yang, Liangyue Tang, Hui Gong

**Affiliations:** ^1^ Department of Cardiology, Jinshan Hospital of Fudan University, Shanghai, China; ^2^ Department of Internal Medicine, Shanghai Medical College, Fudan University, Shanghai, China

**Keywords:** sepsis-induced cardiomyopathy, WGCNA, CIBERSORT, GSEA, immunoreaction

## Abstract

Sepsis-induced cardiomyopathy (SIC), with a possibly reversible cardiac dysfunction, is a potential complication of septic shock. Despite quite a few mechanisms including the inflammatory mediator, exosomes, and mitochondrial dysfunction, having been confirmed in the existing research studies we still find it obscure about the overall situation of gene co-expression that how they can affect the pathological process of SIC. Thus, we intended to find out the crucial hub genes, biological signaling pathways, and infiltration of immunocytes underlying SIC. It was weighted gene co-expression network analysis that worked as our major method on the ground of the gene expression profiles: hearts of those who died from sepsis were compared to hearts donated by non-failing humans which could not be transplanted for technical reasons (GSE79962). The top 25 percent of variant genes were abstracted to identify 10 co-expression modules. In these modules, brown and green modules showed the strongest negative and positive correlation with SIC, which were primarily enriched in the bioenergy metabolism, immunoreaction, and cell death. Next, nine genes (*LRRC39*, *COQ10A*, *FSD2*, *PPP1R3A*, *TNFRSF11B*, *IL1RAP*, *DGKD*, *POR*, and *THBS1*) including two downregulated and seven upregulated genes which were chosen as hub genes that meant the expressive level of which was higher than the counterparts in control groups. Then, the gene set enrichment analysis (GSEA) demonstrated a close relationship of hub genes to the cardiac metabolism and the necroptosis and apoptosis of cells in SIC. Concerning immune cells infiltration, a higher level of neutrophils and B cells native and a lower level of mast cells resting and plasma cells had been observed in patients with SIC. In general, nine candidate biomarkers were authenticated as a reliable signature for deeper exploration of basic and clinical research studies on SIC.

## Introduction

Due to a ununiform definition of sepsis and different mechanisms of disease reporting, the precise incidence data of sepsis remain vague. So, those data are mainly obtained from developed countries with a result showing 2.8 million deaths per year caused by sepsis (Cecconi et al., 2018). Accordingly, as a result of the disparate definition of SIC and some other reasons, the prevalence of SIC is also inaccurate. Sepsis-induced cardiomyopathy with clinical manifestation is defined as an overlapped situation of systolic dysfunction, perfusion failure without systolic dysfunction, and enhanced levels of biomarkers (Hollenberg and Singer, 2021). Many pieces of evidence have shown that the ventricular myocardium is suppressed with a characteristic of diastolic cardiac dysfunction during the occurrence of sepsis (Kakihana et al., 2016). Quite a few factors make contributions to this occurrence including the inflammatory mediator, exosomes, metabolic dysfunction, and oxidative stress (Liu et al., 2017). But, details about gene co-expression related to the pathological process of SIC remain unclear. Thus, elucidating the underlying genes correlated to SIC may be instrumental for in-depth biological and clinical research studies.

As a recently emerging method, weighted gene co-expression network analysis (WGCNA) is a promising mechanism with high efficiency utilized to process the analysis of bioinformatics (Langfelder and Horvath, 2008). In this way, it is available to explore the strongly synergistically altered gene sets and authenticate the strongest related phenotypes via a preset parameter (Nambou et al., 2021). Thus, an increasing number of researchers have applied this technique into a variety of different subjects, especially going viral in pathways and gene identification (Farhadian et al., 2021; Panahi and Hejazi, 2021). Due to its excellent function, WGCNA has also been used in the fields of cardiovascular diseases to identify quite a few potential genes or disease-related pathways successfully, whereas detecting in molecular mechanisms of SIC has not been reported.

To fill the vacancy of this issue, a gene co-expression network had been carried out on account of the GSE79962 dataset, and then two representative modules were identified which showed the most positive and negative relationship with SIC. Different expression genes (DEGs) and the top 10 genes of these two modules were utilized to obtain the hub genes. Then, functional enrichment analysis was performed to find out the roles the interested genes played in the pathological process, and gene set enrichment analysis (GSEA) meant a procession to disclose underlying mechanisms of real hub genes. Moreover, the CIBERSORT method was employed to estimate the infiltrated situation of specific immunocytes among patients with SIC. Quantification of every ROC curve had been carried out to evaluate the accuracy of hub genes as a biomarker.

This research reveals the underlying genes, pathways, and related biological processes of SIC, providing a promising insight into the research and exploration of diseases related to SIC.

## Materials and Methods

### Data Acquisition and Data Handling

The SIC-related datasets were obtained from GSE79962 (https://www.ncbi.nlm.nih.gov/geo/query/acc.cgi?acc=GSE79962), which served as a target set in this study for the following process and to predict the potential hub genes and concerning factors related to SIC. The datasets that we used carried the information about transcriptional profiling of hearts from sepsis-induced death and hearts of unsuccessfully transplanted non-failing human donors for some reasons. A total of 31 samples were contained in datasets: 20 sepsis patients and 11 non-failing donors. Processing and analysis of collected data were achieved by R version 4.0.4 software and making use of the limma package to handle the original data and make them analyzed. After the data processing was completed, 4,336 genes, contained in the dataset GSE79962, showed a prominent expression discrepancy (the top 25% of rank genes), which were used to conduct WGCNA analysis.

### Construction of WGCNA

The R package termed “WGCNA” (Langfelder and Horvath, 2008) was performed to set up the network construction of 4,336 genes obtained from the GSE79962 dataset. Scale independence and mean connectivity were identified via the *x*-axis power value setting of 1–30. Meanwhile, a soft threshold was reasonably selected as the degree of scale independence reached 0.9. In the gene dendrogram, based on the min module size setting to 30 and the application of hierarchical clustering, the genes exhibiting analogical expressive characteristics were sorted into the same modules on the ground of the topological overlap matrix (TOM) dissimilarity. MergeCutHeight referred to the height for cutting the dendrogram in the process of module merging, which was associated with the quantities and accuracy of modules that we finally collected. Setting abline to 0.25, an appropriate quantity of modules was harvested. Finally, the modules containing highly homogenous genes had been merged under a 0.25 MergeCutHeight, and 10 modules were finally harvested.

### Selection of Crucial Modules Associated With SIC

Obtained from hierarchical clustering, modules having close connections with characteristics of SIC would serve as crucial ones to continue our following study. Gene significance (GS), log10 transformation of *p*-value, meant the level of genes compared to clinical phenotypes. Module significance (MS) represented the mean value of module significance across entire module-related genes (Langfelder and Horvath, 2008). Among them, the one showing the maximal modulus of MS referred to the most critical and representative sector involved in SIC.

### Functional Enrichment Analysis Performed on Interested Modules

In order to identify the specific role that genes played in the pathological process, the R package termed “clusterProfiler” was utilized to carry out GO and KEGG enrichment analysis on targets that we extracted from interested crucial modules (Yu et al., 2012).

### Authentication of Candidate Hub Genes

During the utilization process of the weighted co-expression network, connectivity was estimated by the modulus of Pearson’s correlation, referred to a sum of the values that represented the correlation between two genes of all intergenic connections. Intra-module connectivity was defined as the connectivity of individual genes in the separate module by its r values to all other counterparts within the same sector. First, 10 genes with the highest within-module connectivity of the brown module and the green module which exhibited the highest negative and positive relevance with SIC were screened out as our candidate hub genes, respectively. Those candidate genes were regarded as the most potential biomarkers in the respective modules. Next, DEGs were obtained via the limma package with a set of |logFC| ≥1. Then, we got the overlapped intersection of candidate hub genes and DEGs to serve as the hub genes used in the follow-up study, and this process was visualized as a Venn diagram via the online tool “VennDetail” (http://hurlab.med.und.edu:3838/VennDetail/).

### Gene Set Enrichment Analysis

Gene set enrichment analysis (GSEA) for individual hub genes was implemented to make further identification of underlying functions of the interesting hub genes involved in SIC. Our target genes’ expressive level served as a phenotype label while “metric for ranking genes” was based on Pearson correlation. In this process, the R package “clusterProfiler” was used. KEGG sets, the reference sets, were chosen as prior gene sets for functional enrichment analysis. The NOM *p*-value at <0.05 was considered to be statistically enriched.

### Gauging the Immunoreaction of 22 Immunocytes Involved in SIC via CIBERSORT

CIBERSORT, a deconvolution algorithm on composing a proportion of immunocytes based on gene expressions, was utilized to gauge the discrepancy of the expressive level of each set of genes compared to all other genes contained in the sample (Newman et al., 2015; Chen et al., 2018). Through this method, estimated abundances of immunocytes had been assessed by 22 given kinds of immunocytes accompanying with 1,000 permutations. Then, we got a matrix of those immunocytes and visualized this result via an R package termed “ggplot2”. Such discrepancies of immunocyte infiltration of SIC groups compared to reference sets were visualized via an R package termed as “ggpubr”.

### Exploration of the Interaction Between Crucial Genes and Immunocytes

To identify the connection between hub genes, we obtained the immunocytes with significant expression differences, for which Spearman’s rank correlation analysis served as a tool, in the R environment, to achieve our goals, while those results were visualized by the “ggplot2” package.

### Receiver Operating Characteristic Curve Analysis

The receiver operating characteristic (ROC) curve, which meant assessments of abilities of diagnostic tests for a biomarker, was achieved by the “pROC package”. The test sensitivity was set at the *y*-axis while the “1-specificity” which referred to the false-positive rate was set at the *x*-axis. Then, areas under the ROC curves had been calculated so as to quantify ROC curves, and those values of areas were abbreviated to AUC. AUC could serve as an indicator of the abilities for diagnosis. The area’s value of AUC was supposed to be between 1.0 and 0.5. The closer the AUC was to 1.0, the more accurate the diagnosis would be.

### Analyses of Statistics

It was R version 4.0.4 software that served as a tool to achieve all statistical processing and analysis. The significance in statistics of the discrepancies was processed via a non-parametric method or *t*-test depending on different features of data. It was regarded as statistically significant when p was at <0.05.

## Results

### Authentication of Crucial Modules Related to SIC

**FIGURE 1:**
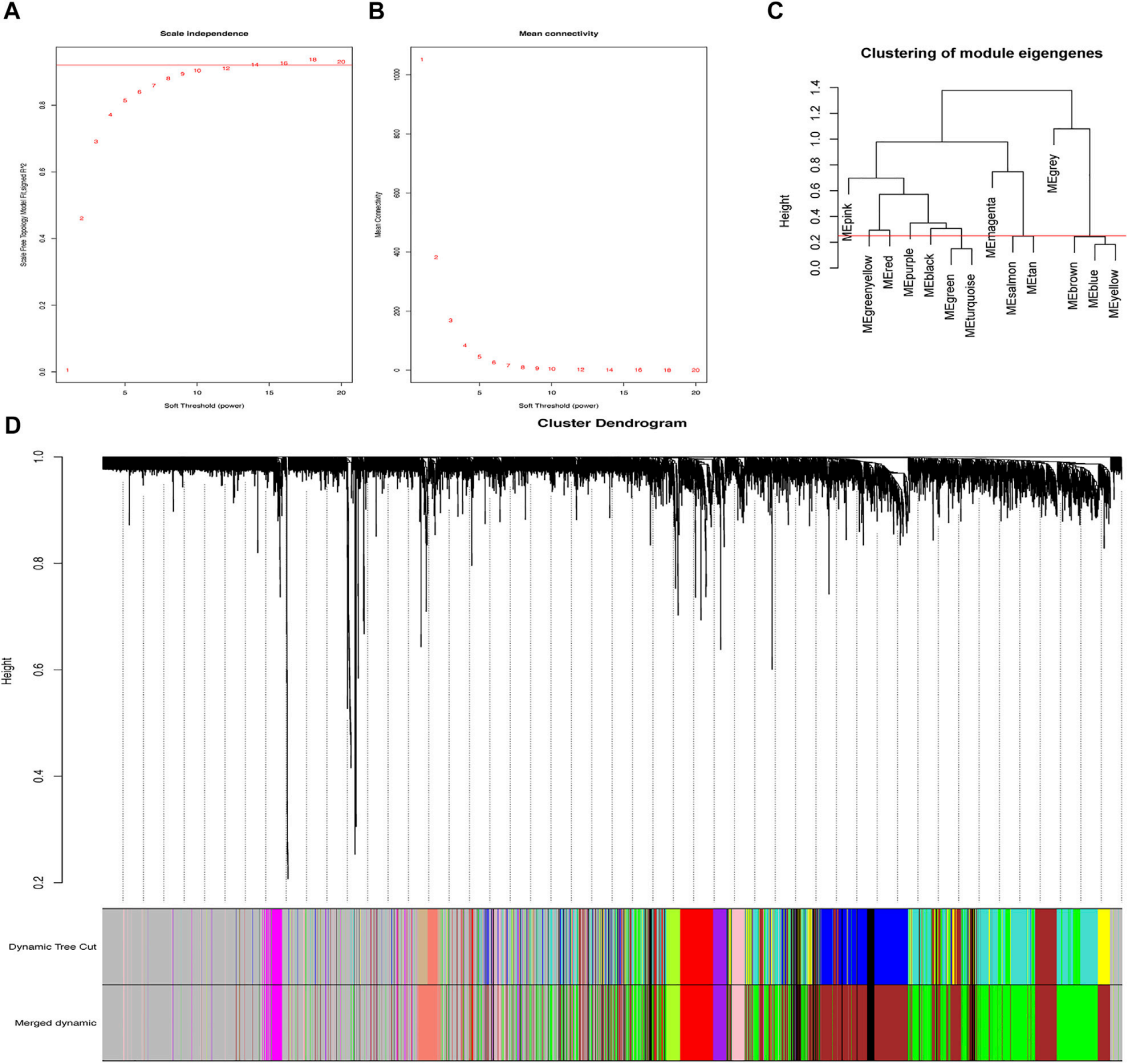
Gene co-expression network. **(A)** Scale-free fit parameters of different soft-thresholding powers. **(B)** According average connectivity of different soft-thresholding powers. **(C)** Cluster of module eigengenes obtained via WGCNA. **(D)** Cluster dendrogram. The different color stands for the different co-expression module we obtained, while black branches mean the genes contained in.

**FIGURE 2:**
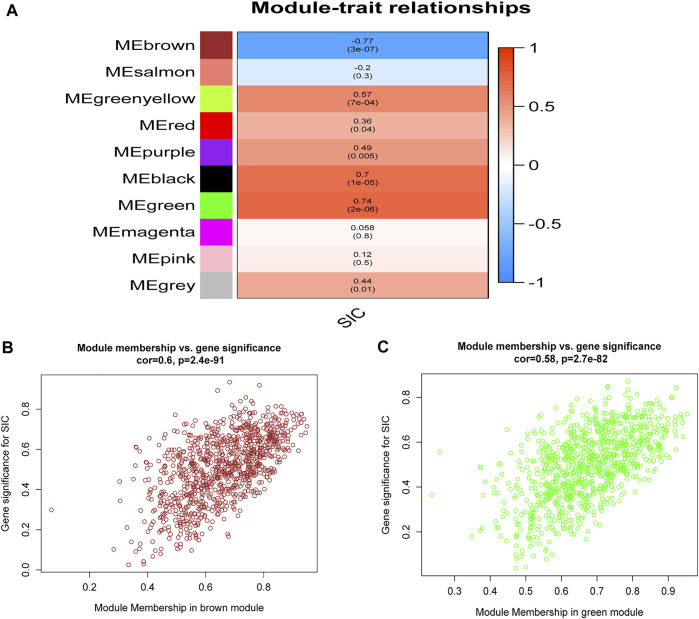
Relationships of module-trait. **(A)** Heatmap showing the connection of the separate module to SIC. Correlation coefficients and *p* values are included in the modules. Red means a strong positive relationship of this module to SIC, while blue represents a strong negative relationship. **(B)** GS for SIC of the brown module and each dot means a gene of this module. **(C)** GS for SIC of the green module and each dot represents a gene in this module. GS: Gene significance. SIC: sepsis-induced cardiomyopathy.

By designing the parameters (soft-thresholding power: 18, scale-free R2: 0.9, and MergeHeightCut: 0.25), 10 candidate modules were obtained (Figures 1A–D). It was shown in module trait correlations analysis that quite a few genes had a relationship with the incidence of SIC (Figure 2A). The brown module (cor = 0.6; p = 2.4e-91, Figure 2B) and the green module (cor = 0.58; p = 2.7e-82, Figure 2C) showed the strongest negative and positive correlation with SIC, respectively. Consequently, the genes of these two promising modules were chosen as candidate genes to conduct further authentication.

### Functional Analysis of Crucial Modules

**FIGURE 3:**
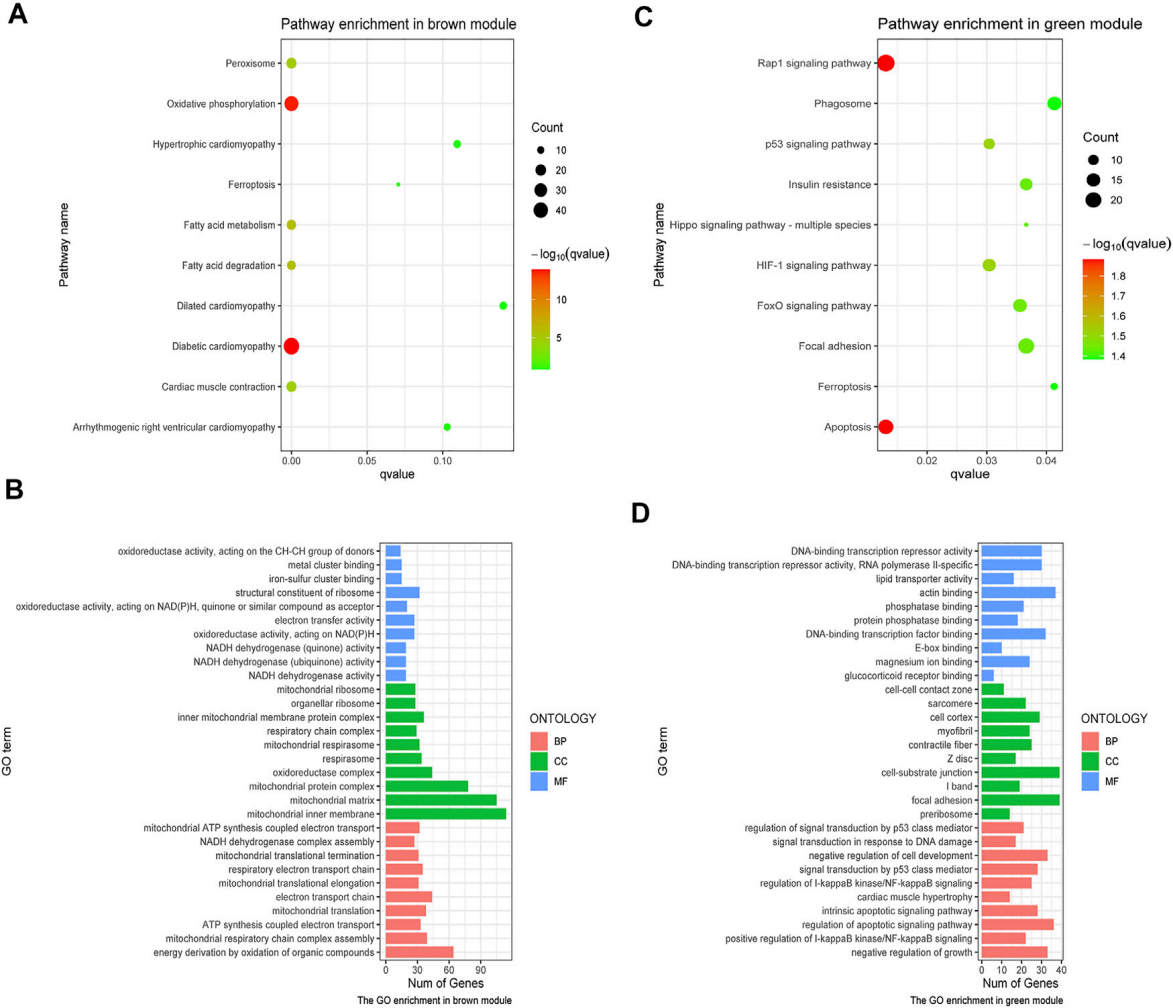
GO and KEGG analysis on crucial modules. **(A)** KEGG analysis on the brown module. **(B)** GO analysis on the brown module. **(C)** KEGG pathway analysis on the green module. **(D)** GO analysis on the green module. The size of the dot represents the count number, while the color means the *p*-value of each pathway.

GO and KEGG analyses were performed on the aforementioned brown and green modules. As a result, it was shown in the KEGG pathway that oxidative phosphorylation, diabetic cardiomyopathy, and fatty acid degradation were the primarily enriched pathways in the brown module (Figure 3A). The GO analysis demonstrated the leading parts of the brown module that included mitochondrial inner membrane, mitochondrial matrix, energy synthesized by organics, and the structural constituent of the ribosome (Figure 3B). In the green module, the top 10 KEGG pathways were chiefly enriched in the rap1 signaling pathway and apoptosis (Figure 3C), while the leading parts demonstrated by the GO terms were actin-binding, cell-substrate junction, focal adhesion and regulation of apoptotic signaling pathway, etc. (Figure 3D). It was obvious that these enriched pathways had a strong relationship with immunoreaction, energetic metabolism, and cell death.

### Authentication of Hub Genes

**FIGURE 4:**
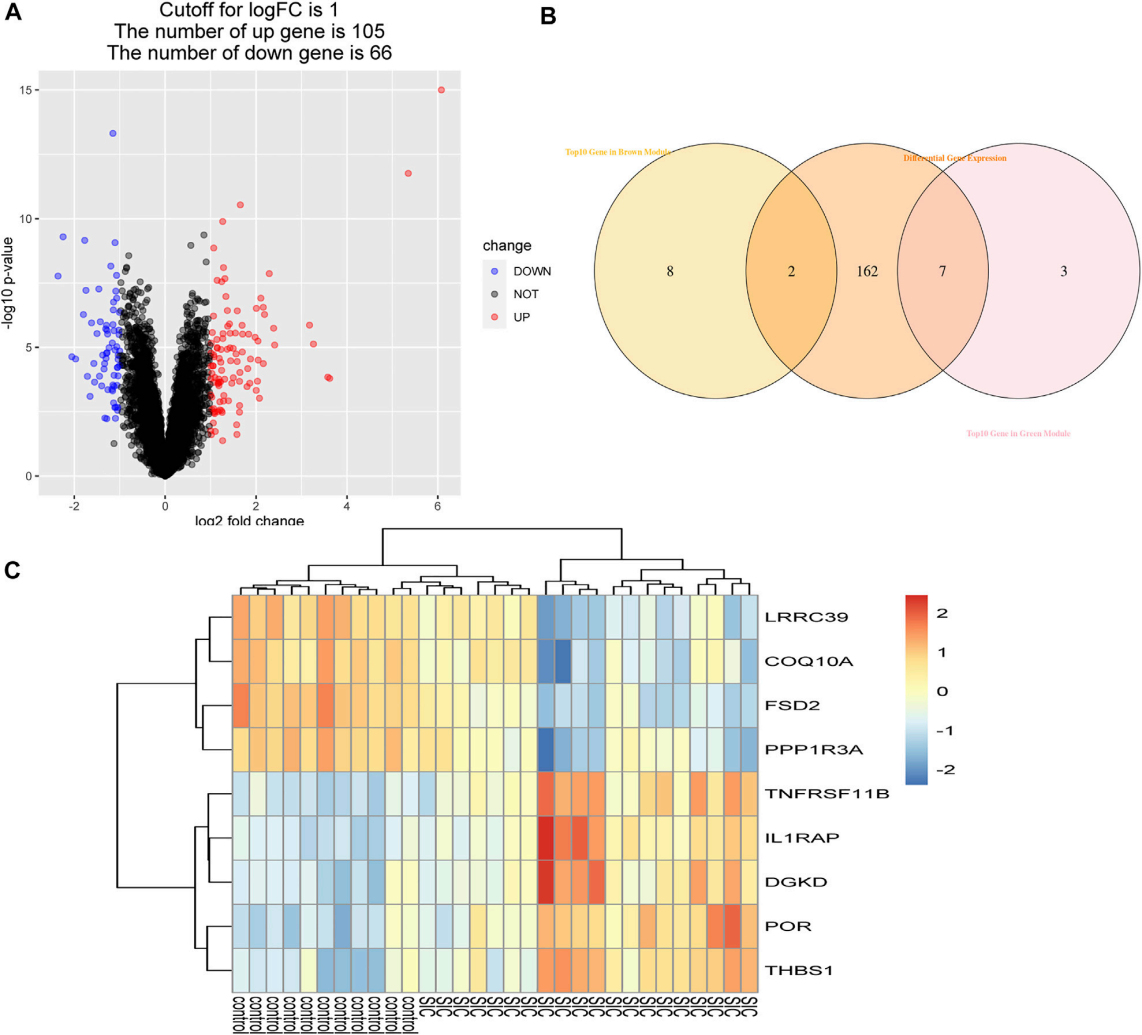
Authentication of hub genes. **(A)** Volcano plot of DEGs. Under the condition of setting |logFC| ≥1, red dots refer to dramatically up-expressive genes, while blue dots represent those downregulated genes. **(B)** Identification of overlapped genes. **(C)** Heatmap of hub genes comparing control groups with SIC groups.

Using the WGCNA, the brown and green modules were obtained as the most relevant module related to SIC, and 20 crucial genes with the highest within-module connectivity were filtered out from those two modules. Then, 171 DEGs were filtered out according to cut-off criteria (|logFC|≥1) as candidate genes containing 105 up-expressed and 66 downregulated genes (Figure 4A). Next, the intersection of 171 DEGs and top 10 genes in the brown module and the green module had been taken (Figure 4B). Thus, those overlapped genes were selected as hub genes including *LRRC39*, *COQ10A*, *FSD2*, *PPP1R3A*, *TNFRSF11B*, *IL1RAP*, *DGKD*, *POR*, and *THBS1* (Figure 4C), which had the strongest positive or negative correlation with SIC. To be more specific, *LRRC39* and *COQ10A* were upregulated, while FSD2, *PPP1R3A*, *TNFRSF11B*, *IL1RAP*, *DGKD*, *POR*, and *THBS1* were downregulated. Further studies would be complemented to figure out the function of these hub genes in the etiology and pathogenesis of SIC.

### Gene Set Enrichment Analysis

**FIGURE 5:**
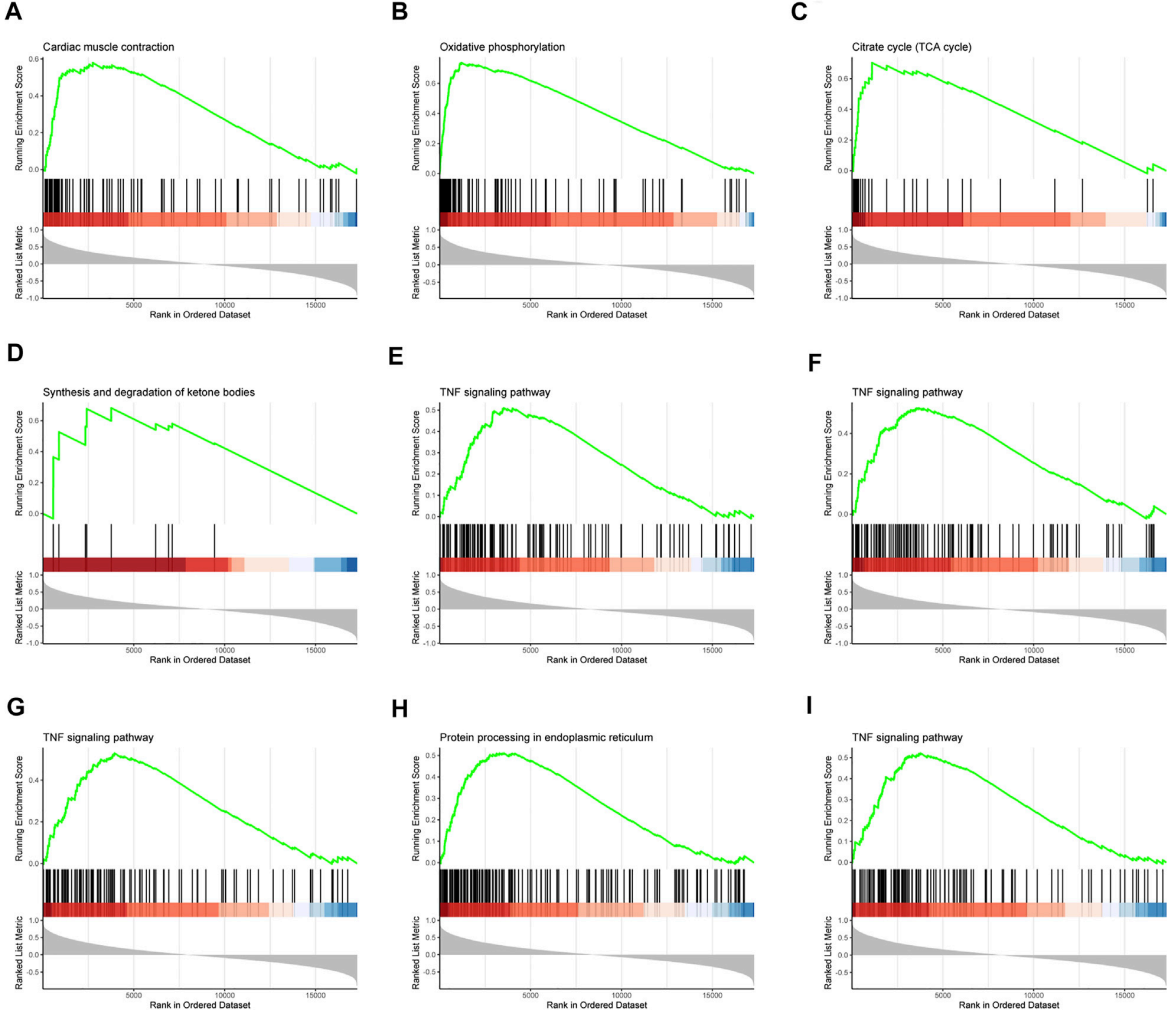
Gene set enrichment analysis (GSEA). The pathway related to hub genes **(A)** *LRRC39*, **(B)** *COQ10A*, **(C)** *FSD2*, **(D)** *PPP1R3A*, **(E)** *TNFRSF11B*, **(F)** *IL1RAP*, **(G)** *DGKD*, **(H)** *POR*, and **(I)** *THBS1*.

Specific effects of our interested genes were explored via GSEA. As what had been shown in the results of GSEA, cardiac muscle contraction, oxidative phosphorylation, TCA cycle, synthesis and degradation of ketone bodies, TNF signaling pathway, and protein processing in endoplasmic reticulum were identified to be strongly related to these nine hub genes (Figures 5A–I). These factors had a close correlation with dysfunction of cardiac metabolism and the necroptosis and apoptosis of cells in SIC. Consequently, these nine hub genes were assumed to exert a sparkly vital contribution to the occurrence and deterioration of SIC.

### Assessment of Immunocytes Infiltration

**FIGURE 6:**
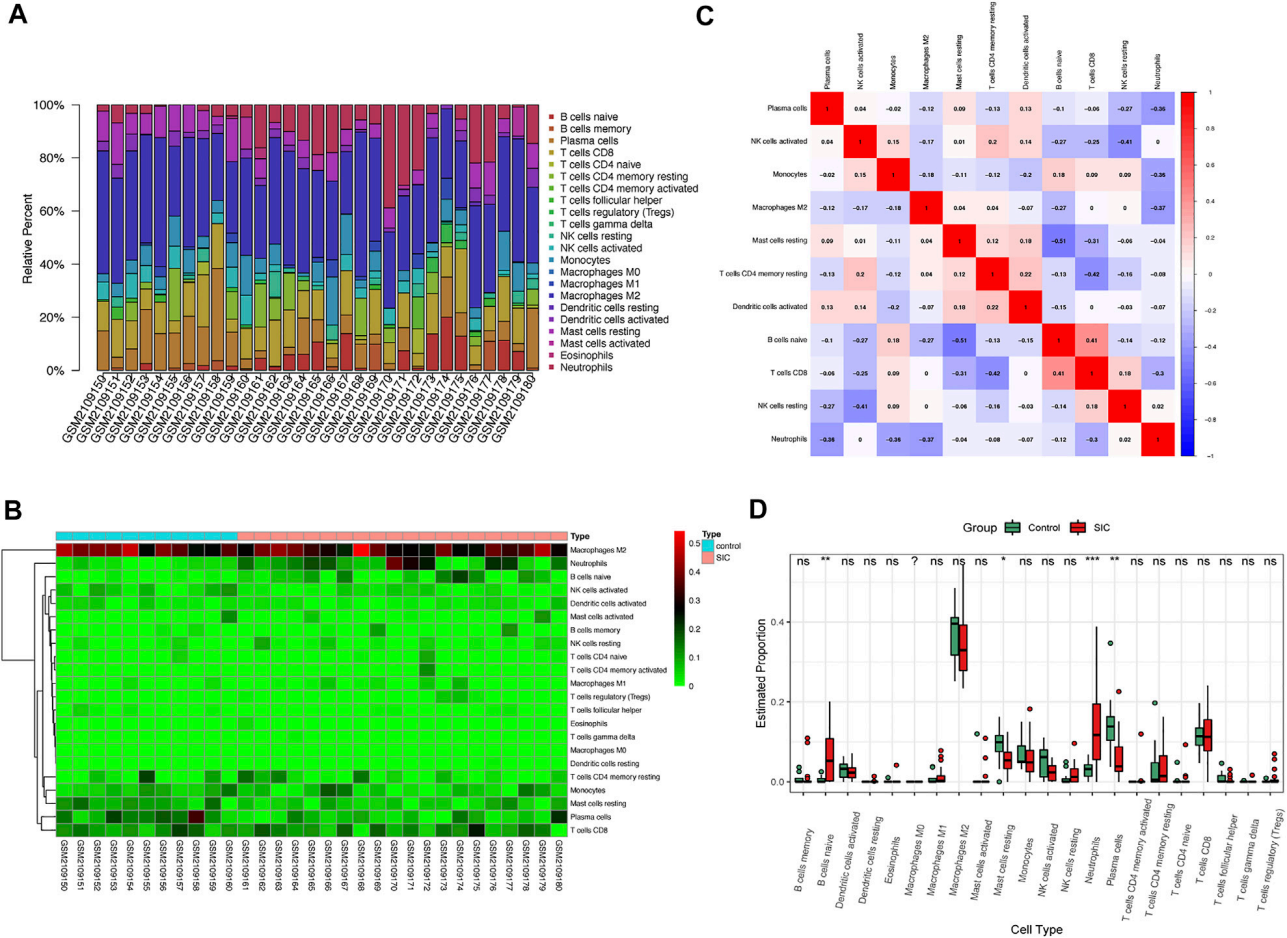
Immunocytes infiltration of control groups compared to SIC groups. **(A)** Proportional values of 22 immunocytes. **(B)** Heatmap of 22 kinds of immunocytes. **(C)** Cor-heatmap showing co-relationship of 11 main immunocytes. **(D)** Differences of infiltration between controls and SIC counterparts. Red boxes stand for the SIC samples. *: *p* < 0.05, **: *p* < 0.01, ***: *p* < 0.001, SIC: sepsis-induced cardiomyopathy.

The aforementioned results demonstrated an evident activation of the immunoreaction. Consequently, the CIBERSORT algorithm was used for the identification of the status of immunocytes of patients suffering from SIC compared to their counterparts. The results clearly showed the composing proportions of given immunocytes in every sample (Figures 6A,B). The cor-heatmap was used to show the co-relationships of 11 main immunocytes (Figure 6C), while the boxplot presented a higher level of neutrophils and B cell native and a lower level of mast cells resting and plasma cells in patients suffering from SIC compared to the counterparts (Figure 6D). Taking all in all, an obvious discrepancy of immune cell infiltration had been shown in the course of SIC.

### The Links of Crucial Genes to Immunocytes

**FIGURE 7:**
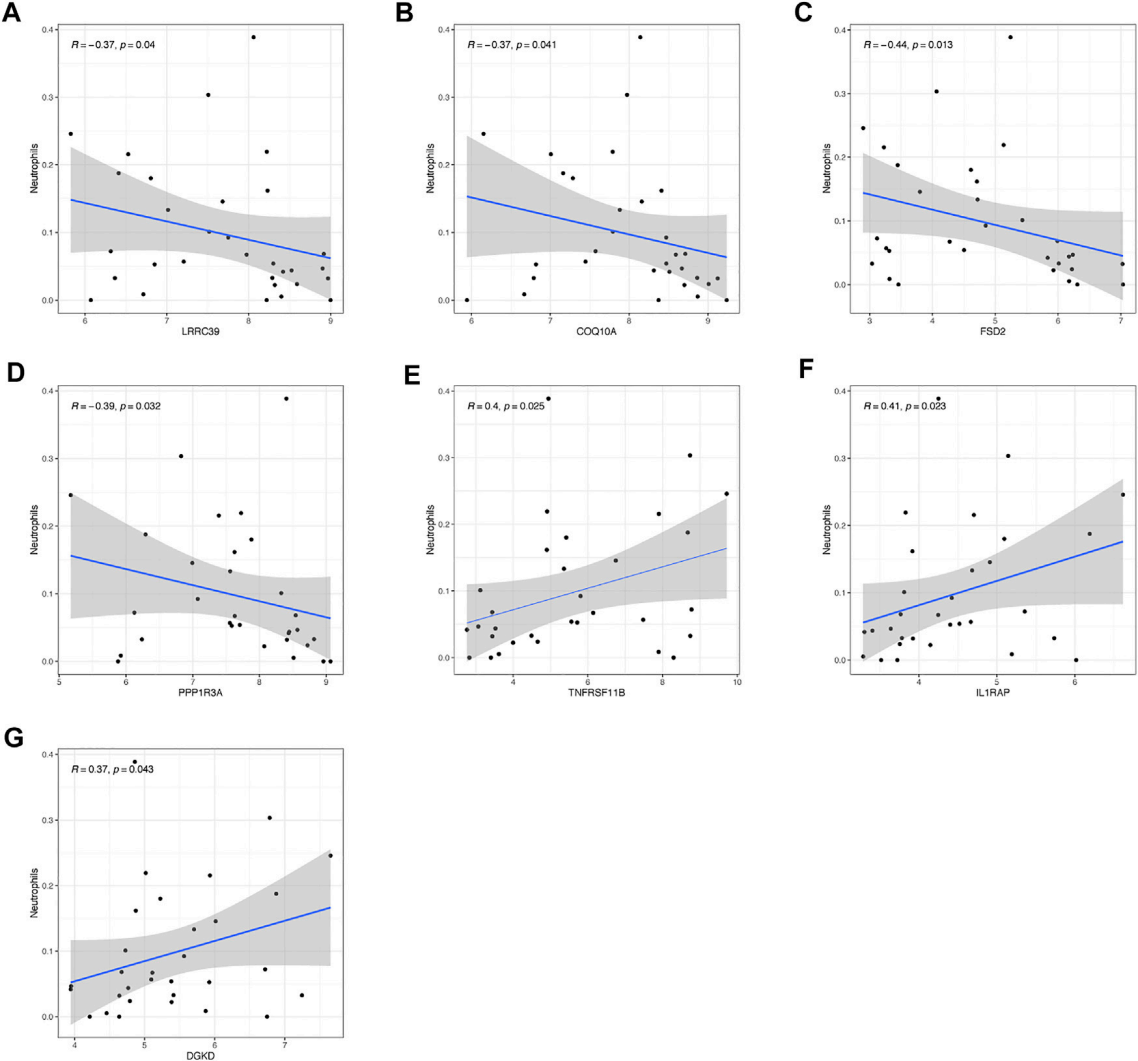
Relationship of hub genes with immunocytes. The connection between neutrophils and **(A)** *LRRC39*, **(B)** *COQ10A*, **(C)** *FSD2*, **(D)** *PPP1R3A*, **(E)** *TNFRSF11B*, **(F)** *IL1RAP*, and **(G)** *DGKD*.

As shown in Figure 7, hub genes showed a strong connection with the effector cell—neutrophils. The analysis uncovered that *LRRC39*, *COQ10A*, *FSD2*, *PPP1R3A*, and *TNFRSF11B* showed a significantly negative correlation with the level of neutrophils. As for *LRRC39*, the value of R was at −0.37 and p was at 0.04. The R-value of *COQ10A* was at −0.37 and p was at 0.041. The R-values of *FSD2* and *PPP1R3A* were −0.44 and −0.39, respectively, while the p was at 0.013 and 0.032. On the contrary, *TNFRSR11B*, *IL1RAP*, and *DGKD* showed a positive correlation with the neutrophils level, which had been shown in the figures. It was noted that *POR* and *THBS1* showed no significant relationship with the effector cells.

### Accuracy of Hub Genes as Diagnostic Genes

**FIGURE 8:**
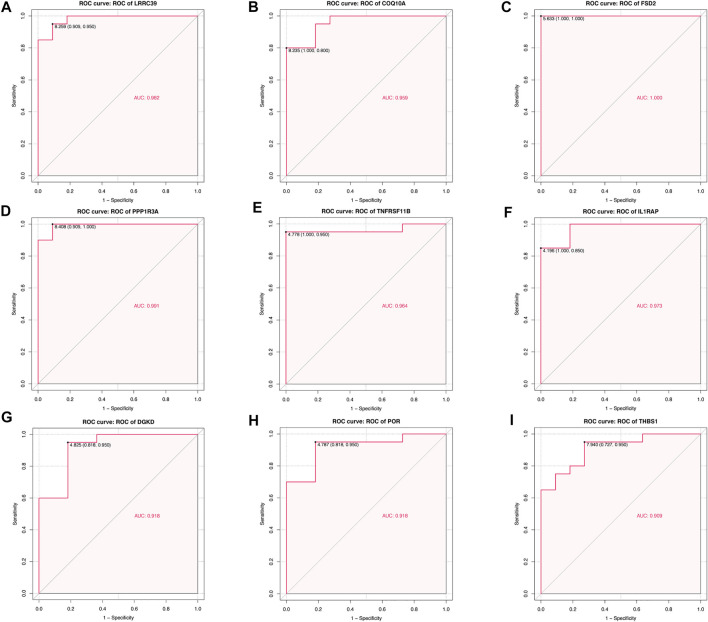
Quantification of ROC curves. Values of AUC for **(A)** *LRRC39*, **(B)** *COQ10A*, **(C)** *FSD2*, **(D)** *PPP1R3A*, **(E)** *TNFRSF11B*, **(F)** *IL1RAP*, **(G)** *DGKD*, **(H)** *POR*, and **(I)** *THBS1*.

For each hub gene obtained, quantifying each ROC curve had been carried out, which could serve as an indicator of the abilities for diagnosis. The AUC of *LRRC39* was valued at 0.982 and *COQ10A* was at 0.959 (Figures 8A,B), and these two genes were figured out to be poor expressive genes in SIC. The AUCs of those over-expressive genes were also shown in the ROC curve figures (Figures 8C–I).

## Discussion

To further explore the underlying genes and mechanisms related to the pathogenesis of SIC, SIC-related gene co-expression networks had been performed via the WGCNA method. As a result, nine genes and several potential biological processes with a strong correlation to SIC had been dug out. Then, the CIBERSORT algorithm was performed for finding out the relationship of immunocytes infiltration between SIC and control counterparts. Finally, the accuracy of those genes as a biomarker had been validated via ROC curves, which furnished evidence of diagnosis and treatment of SIC.

Across 10 modules obtained, the brown and green modules were the most representative components connected with SIC. About the brown one, GO analysis showed that the genes were primarily concerned with mitochondria and the process of energetic metabolism. The KEGG analysis demonstrated oxidative phosphorylation, diabetic cardiomyopathy, fatty acid degradation, and peroxisome that had a strong relation with SIC. These observations were in parallel with the former conclusions. In normal circumstances, the 95% energy in the heart was obtained from oxidative phosphorylation, and it was supplied mainly by fatty acids. However, once SIC occurred, there was a decrease in fatty acid oxidation and significant changes in mitochondrial dysfunction (Wasyluk et al., 2021) which was also associated with the disturbance of oxidative phosphorylation in mitochondria. A peroxisome, which was also called a microbody, was reported to have a relationship with toll-like receptors which could function as an inflammatory signal to reconstruct the energetic metabolism of the heart (Schilling et al., 2011). As far as the green module is concerned, KEGG analysis and GO analysis showed close connections of the rap1 signaling pathway, actin-binding, cell-substrate junction, focal adhesion, and cell death. These results could also be elaborated. Rap1 was a small GTPase controlling several biological processes, such as cell adhesion, the constitution of the cell–cell junction, and actin-binding. Once the actin cytoskeleton was remolded, it would be more vulnerable to suffer deteriorated sepsis (Schnoor et al., 2017). In the process of inflammation, rap1 could also suppress the activity of TNF-α, NF-κB, and pro-inflammatory cytokines (Singh et al., 2021) that was highly related to the occurrence of SIC. As for the apoptosis pathway, it was acknowledged that active caspase-3 and accelerated cardiac apoptosis could be blamed for the development of SIC (Hotchkiss and Nicholson, 2006; Lv and Wang, 2016), which was in accordance with the results of KEGG and GO analysis.

A total of nine hub genes (*LRRC39*, *COQ10A*, *FSD2*, *PPP1R3A*, *TNFRSF11B*, *IL1RAP*, *DGKD*, *POR*, and *THBS1*) were screened out to serve as our candidate genes for biomarker identification including two downregulated and seven upregulated genes. *LRRC39* and *COQ10A* showed a decrease during the occurrence of SIC. *LRRC39* was reported to be concerned with the M-band–associated signaling pathway, which could remold the structure of sarcomeres of cardiac and skeletal muscle. Once it was suppressed *in vivo*, it could result in dysfunction of the heart and cardiomyopathy. So, downregulated *LRRC39* could function similarly to the rap1 signaling pathway mentioned previously to remold the structure resulting in a deterioration of SIC (Will et al., 2010). *COQ10A*, which was primarily expressed in heart and skeletal muscle cells, was necessary for the biosynthetic function of COQ (Tsui et al., 2019). Therefore, the suppression of *COQ10A* might be regarded as an element involved in the dysfunction of oxidative phosphorylation. The latter seven were upregulated genes of which 4 (*TNFRSF11B*, *IL1RAP*, *DGKD*, and *THBS1*) were concerned with the TNF signaling pathway. TNF played a crucial role in the pathological progress of sepsis, which had been regarded as one of the core elements of inflammation (de Pádua Lúcio et al., 2018). The TNF receptor superfamily member 11b (*TNFRSF11B*), also called osteoprotegerin, was a secreted protein and a receptor for the TNF-related apoptosis-inducing ligand, and it was strongly related to NF-κB signaling (Boyce and Xing, 2008). The surface molecule interleukin-1 receptor accessory protein (*IL1RAP*), strongly related to IL-1 signaling, was reported to be an inflammatory mediator (Yu et al., 2020), which could contribute to the expression of inflammation-boosting factors and function in the regulation of cell death (Weber et al., 2010). Moreover, Il-1 β was a potent pro-inflammatory cytokine that strongly induces NF-κB activation. Thus, the upregulation of *IL1RAP* could consequently lead to activation of NF-κB to deteriorate the situation of SIC. Diacylglycerol kinase delta (*DGKD*) was thought to be crucial in cellular signal transduction such as calcium-sensing receptor signaling (Howles et al., 2019). It was also reported to have an impact on metabolism (Chee et al., 2021) and have a strong connection with the immunoreaction (Zhang et al., 2021). So far, the association between this gene and the TNF pathway enriched by GSEA had not been reported, which was still worth a further study. Thrombospondin 1 (*THBS1*) could mediate cell-to-cell and cell-to-matrix reciprocities which were closely related to the progression of sepsis. It had been reported that *THBS1* might be concerned with apoptosis and increase following the activation of the macrophage (Trahtemberg and Mevorach, 2017). Some also confirmed the duality of THBS1 that it could profoundly adjust the function of inflammatory pathways with both pro- and anti-inflammatory effects and validated the molecular interactions that enable *THBS1* to regulate immune cells infiltration (Ramirez et al., 2018). Besides those four genes related to the TNF signaling pathway, the results of GSEA also demonstrated that the rest three genes were correlated with the energetic metabolism. Fibronectin type III and SPRY domain containing 2 (*FSD2*), which was also termed as SPRYD1, was reported to be related with the remolding process of cardiomyocytes (Benson et al., 2017). However, the relationship of FSD2 with SIC and the tricarboxylic acid cycle had not been elucidated, and the information of further research studies were still not available. Protein phosphatase one regulatory subunit 3A (*PPP1R3A*) was capable of regulating the activation of glycogen synthase and phosphorylase kinase (Savage et al., 2008), which might also be connected with the pathological process of SIC. Recently, its function in the development and progress of atrial fibrillation (Alsina et al., 2019) and heart failure (Cordero et al., 2019) had been confirmed. Cytochrome p450 oxidoreductase (*POR*) encoded an oxidoreductase which was indispensable in metabolism (Sugishima et al., 2019). The increase of that element was recognized to be related to cancer recurrences (Pedersen et al., 2019) as well as the regulation of cellular gap junction (Polusani et al., 2011). It could also induce the formation of ROS resulting in a disturbance of apoptosis and necrosis (Navarro-Yepes et al., 2014; He et al., 2017). All these elements mentioned previously could aggravate the development of SIC.

Then, the situation of immunocytes infiltration had been observed in patients with SIC. There were a statistically growing number of neutrophils and B cells native while the number of mast cells resting and plasma cells showed a significant decrease. Neutrophils, a typical immunocyte, could function in both acute and chronic inflammation (Kolaczkowska and Kubes, 2013). It would exert its activities such as facilitating the recruitment of monocytes into inflamed sites and contributing to immune responses in infectious diseases. But in sepsis, it had been reported that the chemotaxis of neutrophils could be interrupted by several elements such as highly concentrated cytokines and chemokines, peroxisome proliferator-activated receptor-γ (PPARγ), and some other elements (Liew and Kubes, 2019). All research led to a conclusion that the dysregulation of neutrophils played a key role in the pathological process. After conducting correlation analysis, we found that seven hub genes showed a significant correlation with this crucial effector cell in SIC—*LRRC39*, *COQ10A*, *FSD2*, *PPP1R3A*, *TNFRSF11B*, *IL1RAP*, and *DGKD*. To be more specific, *LRRC39*, *COQ10A*, *FSD2*, *PPP1R3A*, and *TNFRSF11B* showed a negative relationship with this crucial immunocyte while *IL1RAP* and *DGKD* showed positive trends. It might reveal how those hub genes functioned as immunoregulation molecules to participate in SIC. Therefore, more detailed and in-depth mechanisms of how those genes regulated neutrophils were worth investigating. As for B cells, the significant reduction of plasmacytes, which was matched with the increasing trend of native B cells, played a significant role in sepsis immunosuppression. Not surprisingly, the situation of reduced B cells was observed to be strongly connected with the unsatisfactory outcome of sepsis (Kelly-Scumpia et al., 2011; Monserrat et al., 2013) in *in vivo* studies, and this decrease of B cells was confirmed to be induced by impaired B cells maturation (Duan et al., 2020). It is worth mentioning that the maladjustment of B cells was also blamed for the dysfunction of the heart (Cordero-Reyes et al., 2013; Zouggari et al., 2013; Sarkar and Rafiq, 2019) no matter it was under the function of sepsis or not. Thus, the regulation of B cells maturation was worthy of further study, and this might be used as a potential treatment for SIC. For mast cells, it was not hard to explain the decrease of mast cells resting as the mast cells were activated in face of sepsis. The relationship of mast cells with hypertension, the remolding of collagen fibers (Juliano et al., 2020), and cardiac fibrosis (Kong et al., 2014) had been confirmed, but the function of mastocytes in the pathological process of SIC still required complemented research studies for further elaboration.

Some limitations need to be taken into consideration when explicating those consequences. The size of the data was small for the limited resources, and research studies with larger volumes of data were required to be performed to validate our results. Next, the genes and pathways we obtained required authentication via *in vivo* or *in vitro* studies. Although the area under ROC curves is able to represent the accuracy of one molecule to serve as a diagnostic biomarker, it requires more measures in clinical actions to confirm its feasibility which is concerned with translational medicine. Moreover, cases of co-morbidities in SIC may provide new insights or pieces of evidence for the conclusion we made in this research.

## Conclusion

By and large, we report the promising biomarker genes and signaling pathways of SIC via the WGCNA method. A total of nine hub genes (*LRRC39*, *COQ10A*, *FSD2*, *PPP1R3A*, *TNFRSF11B*, *IL1RAP*, *DGKD*, *POR*, and *THBS1*), *TNF* signaling pathways, rap1 signaling pathway, dysfunction of cytoskeletal structure, immunocytes, and other elements we mentioned previously have been elucidated in this study, and those may provide promising therapeutic methods for the treatment of SIC in the future.

## Data Availability

The original contributions presented in the study are included in the article/Supplementary Material; further inquiries can be directed to the corresponding author.
